# Virtual Reality–Based Training in Chronic Low Back Pain: Systematic Review and Meta-Analysis of Randomized Controlled Trials

**DOI:** 10.2196/45406

**Published:** 2024-02-26

**Authors:** Ran Li, Yinghao Li, Youli Kong, Hanbin Li, Danrong Hu, Chenying Fu, Quan Wei

**Affiliations:** 1 Rehabilitation Medicine Center and Institute of Rehabilitation Medicine West China Hospital Sichuan University Chengdu China; 2 Key Laboratory of Rehabilitation Medicine in Sichuan Province Chengdu China; 3 Department of Orthopedics, Orthopedic Research Institute West China Hospital Sichuan University Chengdu China; 4 National Clinical Research Center for Geriatrics West China Hospital Sichuan University Chengdu China; 5 Aging and Geriatric Mechanism Laboratory West China Hospital Sichuan University Chengdu China

**Keywords:** virtual reality, low back pain, chronic, rehabilitation, exercise

## Abstract

**Background:**

Low back pain is one of the most prevalent pain conditions worldwide. Virtual reality–based training has been used for low back pain as a new treatment strategy. Present evidence indicated that the effectiveness of virtual reality–based training for people with chronic low back pain is inconclusive.

**Objective:**

This study conducted a meta-analysis to evaluate the immediate- and short-term effects of virtual reality–based training on pain, pain-related fear, and disability in people with chronic low back pain.

**Methods:**

We searched the PubMed, Embase, Web of Science, PEDro, CENTRAL, and CINAHL databases from inception until January 2024. Only randomized controlled trials assessing the effects of virtual reality–based training on individuals with chronic low back pain were selected. The outcomes were focused on pain, pain-related fear measured by the Tampa Scale of Kinesiophobia, and disability measured by the Oswestry Disability Index. The immediate term was defined as the immediate period after intervention, and the short term was defined as 3 to 6 months after intervention. The Cochrane Risk of Bias tool and the GRADE (Grading of Recommendations, Assessment, Development and Evaluation) approach were used to evaluate the quality of the methodology and evidence, respectively.

**Results:**

In total, 20 randomized controlled trials involving 1059 patients were eligible for analysis. Virtual reality–based training showed significant improvements in pain (mean difference [MD] –1.43; 95% CI –1.86 to –1.00; *I*^2^=95%; *P*<.001), pain-related fear using the Tampa Scale of Kinesiophobia (MD –5.46; 95% CI –9.40 to 1.52; *I*^2^=90%; *P*=.007), and disability using the Oswestry Disability Index (MD –11.50; 95% CI –20.00 to –3.01; *I*^2^=95%; *P*=.008) in individuals with chronic low back pain immediately after interventions. However, there were no significant differences observed in pain (*P*=.16), pain-related fear (*P*=.10), and disability (*P*=.43) in the short term.

**Conclusions:**

These findings indicated that virtual reality–based training can be used effectively for individuals with chronic low back pain in the immediate term, especially to reduce pain, alleviate pain-related fear, and improve disability. However, the short-term benefits need more high-quality trials to be demonstrated.

**Trial Registration:**

PROSPERO CRD42021292633; http://tinyurl.com/25mydxpz

## Introduction

Low back pain is a common musculoskeletal symptom influenced by complex interactions among biological, psychological, and social factors [1-3]. With more than 568 million people experiencing low back pain globally, it has become the leading cause of years lived with disability worldwide [4,5]. Low back pain is defined by the location of pain, typically between the lower rib margins and the buttock creases [6]. Once the symptom persists for more than 3 months, it can be considered as chronic low back pain. Chronic low back pain may be accompanied by fear avoidance and dysfunction. Research has suggested that individuals who hold negative beliefs about their pain or their condition may experience an exaggerated fear of pain and the potential negative consequences of their symptoms. This fear can lead to a cycle of catastrophizing thoughts, pain-related fear, and avoidance of movements that they perceive as potentially painful or harmful [7]. Furthermore, fear avoidance and catastrophizing thoughts are considered catalysts for chronicity, resulting in prolonged recovery and increased disability rates [8].

Given the impaired physical function, quality of life, and even social participation from chronic low back pain [9], it is a global priority to establish an effective treatment [6,10,11]. Multicomponent exercises are frequently prescribed by physicians for chronic low back pain, as recommended by clinical practice guidelines and established by randomized controlled trials (RCTs) [12-16]. Exercises encompass a diverse set of components including specific activities, postures, or movements (or all) [17]. It is reported that exercises may benefit patients with chronic low back pain by improving muscle strength and movement and enhancing postural musculature, stability, and coordination, or a combination of these factors [18]. Among the various exercises available, virtual reality–based training is becoming increasingly popular for the treatment of chronic low back pain [19-22]. It refers to digital training via computer-generated realities implemented with stereoscopic displays [23,24]. In addition, virtual reality–based training has been shown to reduce the focus on pain by dividing attention to tasks [25,26] and increase the motivation of movement through progressive achievement [27-29].

Two systematic reviews suggested that virtual reality–based training had a positive effect on improving pain intensity [30] and fear avoidance [31] in people with low back pain, while other systematic reviews indicated that the effectiveness of virtual reality–based training was inconclusive [32,33]. To our knowledge, evidence on the immediate-, short-, and long-term benefits of virtual reality–based training in patients with chronic low back pain does not exist as well. The evidence on the potential effectiveness of virtual reality–based training for chronic low back pain is still controversial and deficient. Therefore, we conducted this systematic review and meta-analysis to evaluate the immediate-term and short-term efficacy of virtual reality–based training for chronic low back pain.

## Methods

### Design

This systematic review and meta-analysis followed the PRISMA (Preferred Reporting Items for Systematic Reviews and Meta-Analyses) [34] and PRISMA 2020 guidelines (Multimedia Appendix 1) [35] and was performed following a protocol registered in PROSPERO (CRD42021292633).

### Search Strategy

Two reviewers (RL and YL) independently searched the PubMed, Embase, Web of Science, PEDro, CENTRAL, and CINAHL databases from inception until January 2024. Low back pain and virtual reality were searched as keywords. The full search strategy is described in Multimedia Appendix 2. The bibliographic references of the included studies and previous systematic reviews were also checked to identify additional trials.

### Study Selection

Studies concerning the effects of virtual reality involving individuals with low back pain were included in this systematic review. The inclusion criteria were as follows: (1) RCTs of parallel groups, (2) participants experiencing chronic low back pain, (3) virtual reality as intervention, (4) studies assessed clinical outcomes (eg, pain, pain-related fear, or disability), and (5) duration of intervention ≥8 sessions. The exclusion criteria were as follows: (1) reviews, case reports, and conference abstracts; (2) studies without enough information for data analysis; and (3) studies not written in English. The detailed inclusion and exclusion criteria are shown in Textbox 1.

**The inclusion and exclusion criteria.**

**Inclusion criteria**

- Paper type: randomized controlled trials of parallel groups
- Study subjects: participants with chronic low back pain (3 months or more)
- Interventions: virtual reality–based training as intervention alone or in combination with physical therapy, irregular of virtual reality devices; the duration of intervention is ≥8 sessions
- Outcomes: at least 1 of the following outcome measurements: pain (Visual Analog Scale, Numerical Rating Scale, and Defense and Veterans Pain Rating Scale), pain-related fear (Tampa Scale of Kinesiophobia), or disability (Oswestry Disability Index)
- Language: written in English

**Exclusion criteria**

- Paper type: reviews, case reports, and conference abstracts
- Study subjects: participants with other pain or low back pain lasting for less than 3 months
- Interventions: no interventions involved virtual reality
- Outcomes: without the required outcomes or no available data and not enough information for analysis
- Language: non-English publications

### Outcome Measures

The outcomes were focused on pain, pain-related fear, and disability. The pain intensity was measured by the Visual Analog Scale, Numerical Rating Scale, and Defense and Veterans Pain Rating Scale, which have demonstrated strong validity and reliability in clinical and research settings [36-39]. The pain-related fear was measured by the Tampa Scale of Kinesiophobia, which is frequently used in patients with back pain [40,41]. As for the functional disability, the Oswestry Disability Index (ODI) was used [42].

### Data Extraction

Two reviewers (RL and YL) independently extracted the main information for the included studies using a standard extraction spreadsheet (Microsoft Excel, Microsoft Corp). A third reviewer (YK) was consulted if the initial reviewers (RL and YL) disagreed. The detailed characteristics of the selected studies were summarized, which included study characteristics (author, year of publication, country, sample size, and follow-up points), population characteristics (gender, age, and pain duration), intervention characteristics (type, frequency, and duration), and outcome measurements (pain, pain-related fear, and disability).

### Quality Assessment

The quality of RCTs was assessed using the Cochrane Risk of Bias tool [43], which consists of 5 domains and an overall judgment. The 5 domains include randomization process, deviations from intended interventions, missing outcome data, measurement of the outcome, and selection of the reported result. Based on the answers to a series of signaling questions, the judgment options in each domain consist of “low risk of bias,” “some concerns,” and “high risk of bias.” Two independent researchers (RL and HL) assessed the quality of each eligible study. Disagreements in the data were settled by consensus.

We used the GRADE (Grading of Recommendations, Assessment, Development and Evaluation) approach to assess the quality of evidence, which is rated as high, moderate, low, and very low [44]. The quality of RCTs is initially considered high and then downgraded based on risk of bias, imprecision, inconsistency, indirectness, and publication bias [45-49].

### Statistical Analysis

This meta-analysis was conducted using Review Manager (version 5.30; Cochrane Collaboration), and all extracted data were input and checked by the reviewers (RL, YL, and YK). Mean differences (MDs) were calculated with the 95% CIs using the inverse variance method, when the outcomes were evaluated with the same scale. The chi-square test and inconsistency (*I*^2^) were used to calculate statistical heterogeneity. The random effect model was used when *I*^2^>50%; otherwise, the fixed-effect model was used. Statistical differences by meta-analysis were identified as *P*<.05. Furthermore, the minimal clinically important difference (MCID) was taken into consideration in this study, and the standard was established according to a previous report.

Subgroup analyses comparing the efficacy of virtual reality on pain, pain-related fear, and disability were performed for immediate-term and short-term outcomes separately. The immediate term was defined as the immediate period after intervention, and the short term was defined as 3 to 6 months after intervention.

## Results

### Study Selection and Characteristics

**Figure 1:**
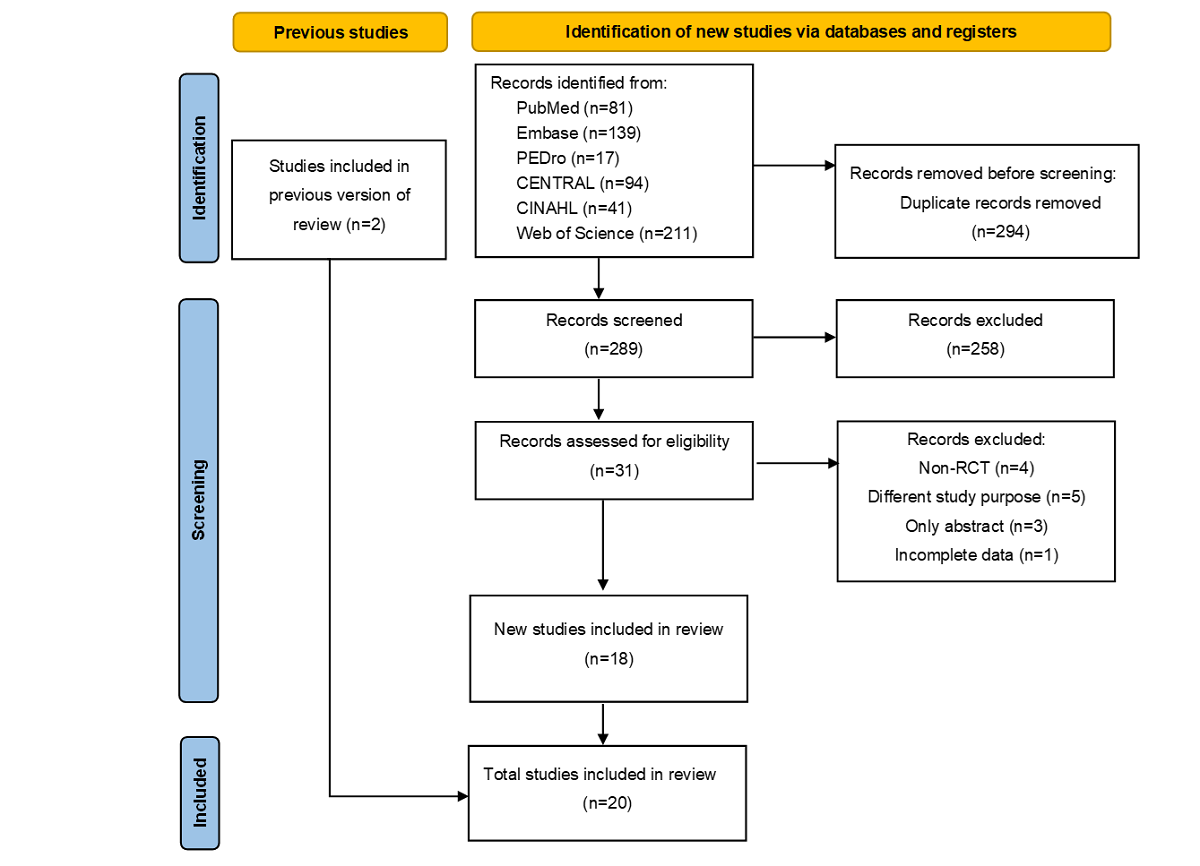
Flowchart showing the study selection process. RCT: randomized controlled trial.

The initial search procedure yielded 583 records in total, 294 of which were removed due to duplication. After the screening process, 20 studies were selected for this systematic review and meta-analysis (Figure 1). The 20 papers included 1059 individuals diagnosed with chronic low back pain. The characteristics (sample size, gender, interventions, exposures, and outcome measurements) of the involved studies were summarized (Table 1). All studies had an RCT design: 7 used a 3-arm parallel-group design [21,50-55], and 1 used a 4-arm parallel-group design [56]. Of the included papers, 4 studies involved only female patients [20,22,57,58], and 6 studies involved only male patients [52-56,59].

**Table 1:** Characteristics of the included studies.

Study (year)	Country	Sample size	Gender	Mean age (years)	Pain duration (months)	Intervention	Exposure	Outcome measures	Follow-up points
Afzal et al (2022) [19]	Pakistan	Exp^a^: 42 Con^b^: 42	Male: 28 Female: 56	Exp: 38.2 Con: 37.5	—^c^	Exp: Virtual reality–based exercises via kinetic exergames+physical therapy Con: Back strengthening exercises+physical therapy	12 sessions; 3 times per week	VAS^d^; ODI^e^	4 weeks
Eccleston et al (2022) [50]	Finland	Exp1: 14 Exp2: 17 Con: 11	Male: 5 Female: 37	Exp1: 55.14 Exp2: 52.76 Con: 57.09	>6	Exp1: Virtual reality–based cognitive behavioral intervention via Oculus Quest and Touch virtual reality headset and handheld controllers Exp2: Viewed text-based cognitive behavioral intervention via Oculus Quest and Touch virtual reality headset Con: Standard care	30 sessions; 5 times per week; 15-60 minutes per session	NRS^f^; ODI; PROMIS^g^ 6b; TSK^h^; pain medications; EuroQoL-5D-5L^i^; adverse events; PGIC^j^; Game Experience Questionnaire	9 weeks, 5 months
Garcia et al (2021) [60]	United States	Exp: 94 Con: 94	Male: 43 Female: 144	Exp: 52.1 Con: 51.3	≥6	Exp: Virtual reality–based cognitive behavioral therapy via Pico G2 4K head-mounted virtual reality device (3D visual displays) Con: Sham virtual reality–based cognitive behavioral therapy via Pico G2 4K head-mounted virtual reality device	56 sessions; 7 times per week; 2-16 minutes per session	DVPRS^k^; DVPRS-II; PSEQ^l^-2; PGIC; PROMIS 6b and 6a; PCS^m^; CPAQ-8^n^	8 weeks, 3 months, 6 months
Groenveld et al (2023) [61]	Netherlands	Exp: 20 Con: 20	Male: 7 Female: 33	Exp: 51 Con: 52	Exp: 5 Con: 4	Exp: Virtual reality–based exercises via an Oculus Go head-mounted display Con: Daily life routines	60 sessions; 3 times per day; 10-30 minutes per session	SF-12^o^; VAS; PCS; HADS^p^; ODI; PCCL^q^; NEADL^r^; BPI^s^	4 weeks, 4 months
Kim et al (2014) [20]	South Korea	Exp: 15 Con: 15	Male: 0 Female: 30	Exp: 44.33 Con: 50.46	—	Exp: Virtual reality–based yoga program via Wii Fit activities Con: Trunk stabilizing exercise+physical therapy program	12 sessions; 3 times per week; 30 minutes per session	VAS; pressure algometry; ODI; RMDQ^t^; FABQ^u^	4 weeks
Kim et al (2020) [62]	South Korea	Exp: 24 Con: 24	Male: 26 Female: 22	Exp: 26.0 Con: 28.79	Exp: 58.22 Con: 101.55	Exp: Virtual reality–based simulated horseback riding Con: Stabilization exercise via suspension	16 sessions; 2 times per week; 46 minutes per session	NRS; ODI; RMDQ; FABQ	4 weeks, 8 weeks, 6 months
Li et al (2021) [51]	China	Exp1: 11 Exp2: 12 Con: 11	Male: 9 Female: 25	Exp1: 21.91 Exp2: 23.75 Con: 25.36	Exp1: 30.18 Exp2: 38.83 Con: 49.82	Exp1: Virtual reality–based Fruit Ninja game via Kinect Xbox 360+magnetic therapy Exp2: Ultrasound-guided abdominal drawing-in maneuver training+magnetic therapy Con: Conventional thermal magnetic therapy	10 sessions; 5 times per week; 30 minutes per session	VAS; ODI; sEMG^v^	2 weeks
Meinke et al (2022) [63]	Switzerland	Exp: 13 Con: 14	Male: 10 Female: 17	Exp: 40.14 Con: 40.85	—	Exp: Virtual reality–based exergame via 2 inertial measurement units Con: Daily life routines	9 sessions; 3 times per week; 20 minutes per session	NRS; TSK; RMDQ; WHOQOL-Bref^w^	3 weeks
Monteiro-Junior et al (2015) [57]	Brazil	Exp: 16 Con: 14	Male: 0 Female: 30	68	—	Exp: Virtual reality–based physical training via Nintendo Wii-motion and WBBx+core and strength training Con: Core and strength training	24 sessions; 3 times per week; 90 minutes per session	NRS; WBB; sit-to-stand test; POMS^y^	8 weeks
Nambi et al (2020) [54]	Saudi Arabia	Exp1: 15 Exp2: 15 Con: 15	Male: 45 Female: 0	Exp1: 21.25 Exp2: 20.23 Con: 20.78	Exp1: 4.1 Exp2: 4.1 Con: 4.3	Exp1: Virtual reality–based-balance training via Pro-Kin system PK 252 N Exp2: Balance training via Swiss ball Con: Conventional balance training via active isotonic and isometric exercise	20 sessions; 5 times per week; 30 minutes per session	VAS; player wellness; sprint performance; jump performance	4 weeks, 8 weeks, 6 months
Nambi et al (2021) [55]	Saudi Arabia	Exp1: 20 Exp2: 20 Con: 20	Male: 60 Female: 0	Exp1: 21.45 Exp2: 21.39 Con: 20.97	Exp1: 4.8 Exp2: 5.2 Con: 4.9	Exp1: Virtual reality training via firing game Exp2: Core stabilization training via therapeutic ball Con: Traditional active balance exercise	20 sessions; 5 times per week; 30 minutes per session	NRS; physical fitness index; sprint performance; jump performance	4 weeks, 8 weeks, 6 months
Nambi et al (2021) [53]	Saudi Arabia	Exp1: 18 Exp2: 18 Con: 18	Male: 54 Female: 0	Exp1: 22.3 Exp2: 21.4 Con: 21.9	Exp1: 5.4 Exp2: 5.3 Con: 5.5	Exp1: Virtual reality training+hot pack therapy+ultrasound Exp2: Isokinetic training+hot pack therapy+ultrasound Con: Conventional core training+hot pack therapy+ultrasound	20 sessions; 5 times per week; 30 minutes per session	VAS; TSK; blood serum level of stress hormones	4 weeks, 6 months
Nambi et al (2021) [52]	Saudi Arabia	Exp1: 20 Exp2: 20 Con: 20	Male: 60 Female: 0	Exp1: 23.2 Exp2: 22.8 Con: 23.3	Exp1: 5.8 Exp2: 5.2 Con: 5.4	Exp1: Virtual reality training+hot pack therapy+ultrasound Exp2: Isokinetic training+hot pack therapy+ultrasound Con: Conventional core training+hot pack therapy+ultrasound	20 sessions; 5 times per week; 30 minutes per session	VAS; TSK; blood serum level of stress hormones	4 weeks, 6 months
Oh et al (2014) [56]	South Korea	Exp1: 9 Exp2: 9 Exp3: 10 Con: 9	Male: 37 Female: 0	Exp1: 20.7 Exp2: 20.56 Exp3: 20.33 Con: 20.44	Exp1: 6.38 Exp2: 6.21 Exp3: 7.57 Con: 6.75	Exp1: Virtual reality training via horse simulator machine for 10 minutes Exp2: Virtual reality training via horse simulator machine for 20 minutes Exp3: Virtual reality training via horse simulator machine for 30 minutes Con: Daily life routines	40 sessions; 5 times per week; 15-35 minutes per session	VAS; body composition; isokinetic trunk and hip extension or flexion and hip abduction or adduction	8 weeks
Park et al (2013) [21]	South Korea	Exp1: 8 Exp2: 8 Con: 8	—	Exp1: 44.12 Exp2: 43.37 Con: 44.12	Exp1: 17.0 Exp2: 16.0 Con: 18.75	Exp1: Virtual reality–based training via Nintendo Wii program+physical therapy Exp2: Lumbar stabilization exercise+physical therapy Con: Physical therapy (eg, hot pack, interferential current therapy, and deep heat with ultrasound)	24 sessions; 3 times per week; 80 minutes per session	VAS; isometric lifting strength for back strength; 1-legged Stand Test for balance ability; RAND-36^z^; SF-36^aa^	8 weeks
Park et al (2020) [58]	South Korea	Exp: 40 Con: 40	Male: 0 Female: 80	Exp: 71.35 Con: 72.05	Exp: 23.61 Con: 22.10	Exp: Virtual reality–based training via equestrian simulator Con: Sitting on the equestrian simulator	36 sessions; 3 times per week; 30 minutes per session	VAS; ODI; body composition; isokinetic trunk extension and flexion; spinal alignment	12 weeks
Yalfani et al (2022) [22]	Japan	Exp: 13 Con: 12	Male: 0 Female: 25	Exp: 68 Con: 67.08	>6	Exp: Virtual reality–based training via Xbox Kinect headset Con: Daily life routines	24 sessions; 3 times per week; 30 minutes per session	VAS; SF-36; FRI^ab^; BBS^ac^	2 weeks
Yilmaz Yelvar et al (2017) [64]	Iran	Exp: 22 Con: 22	Male: 16 Female: 28	Exp: 46.27 Con: 52.81	Exp: 5.27 Con: 7.45	Exp: Virtual walking task via Vita Digital Productions+physical therapy Con: Physical therapy (eg, hot pack, TENSad, deep heat with ultrasound, and therapeutic exercises)	10 sessions; 5 times per week	VAS; TSK; ODI; Nottingham Health Profile; TUG^ae^; 6MWT^af^; single-leg balance test	2 weeks
Yoo et al (2014) [59]	South Korea	Exp: 24 Con: 23	Male: 47 Female: 0	Exp: 20.44 Con: 20.7	Exp: 9.41 Con: 8.35	Exp: Virtual reality–based training via horse simulator Con: Daily life routines	24 sessions; 3 times per week; 20-50 minutes per session	VAS; body composition; isokinetic trunk strength	8 weeks
Zadro et al (2019) [65]	Turkey	Exp: 30 Con: 30	Male: 29 Female: 31	Exp: 68.8 Con: 67.8	>3	Exp: Video game home-based exercise via Nintendo Wii U console Con: Daily life routines	24 sessions; 3 times per week; 60 minutes per session	NRS; TSK; PSEQ; 3-item questionnaire; Rapid Assessment of Physical Activity questionnaire; PSFS^ag^; RMDQ; 16-item Falls Efficacy Scale-International Questionnaire	8 weeks

^a^Exp: experimental group.

^b^Con: control group.

^c^Not available.

^d^VAS: Visual Analog Scale.

^e^ODI: Oswestry Disability Index.

^f^NRS: Numerical Rating Scale.

^g^PROMIS: Patient-Reported Outcomes Measurement Information System.

^h^TSK: Tampa Scale of Kinesiophobia.

^i^EuroQoL-5D-5L: European Quality of Life 5-dimension, 5-level scale.

^j^PGIC: Patient’s Global Impression of Change.

^k^DVPRS: Defense and Veterans Pain Rating Scale.

^l^PSEQ: Pain Self-Efficacy Questionnaire.

^m^PCS: Pain Catastrophizing Scale.

^n^CPAQ-8: Chronic Pain Acceptance Questionnaire.

^o^SF-12: 12-item Short-Form Health Survey.

^p^HADS: Hospital Anxiety and Depression Scale.

^q^PCCL: Pain Coping and Cognition List.

^r^NEADL: Nottingham Extended Activities of Daily Living.

^s^BPI: Brief Pain Inventory.

^t^RMDQ: Roland Morris Disability Questionnaire.

^u^FABQ: Fear Avoidance Beliefs Questionnaire.

^v^sEMG: surface electromyography.

^w^WHOQOL-Brief: World Health Organization Quality of Life Questionnaire-short version.

^x^WBB: Wii Balance Board.

^y^POMS: Profile of Mood States.

^z^RAND-36: RAND-36 Health Status Inventory.

^aa^SF-36: 36-item Short-Form Health Survey.

^ab^FRI: Fall Risk Index.

^ac^BBS: Biodex Balance System.

^ad^TENS: transcutaneous electrical nerve stimulation.

^ae^TUG: Timed Up and Go Test.

^af^6MWT: 6-Minute Walk Test.

^ag^PSFS: Patient-Specific Functional Scale.

### Risk of Bias

**Figure 2:**
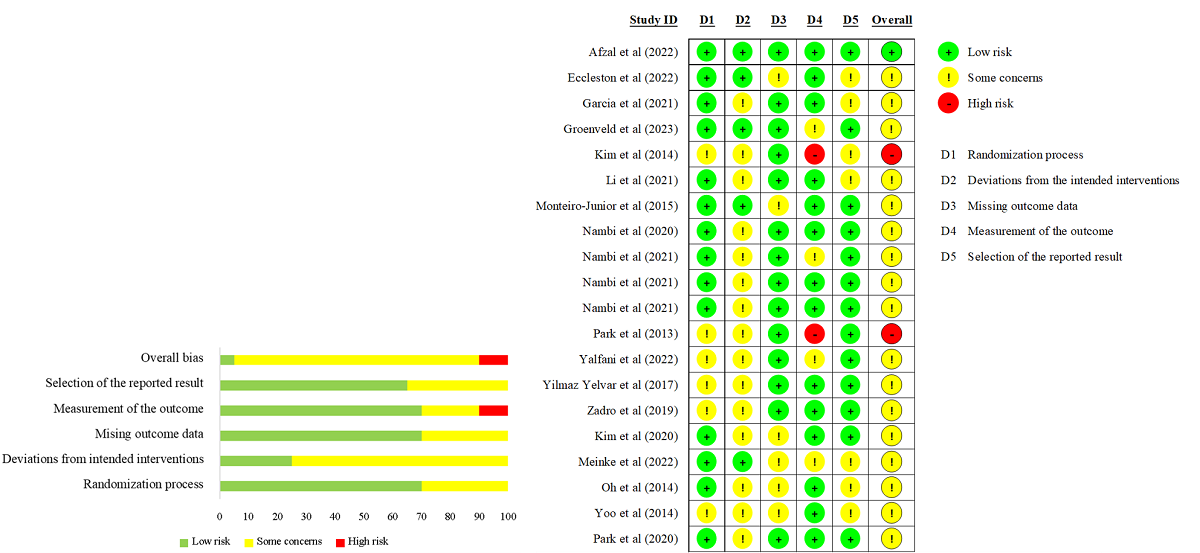
Risk-of-bias graph and summary.

In total, 20 studies were considered as having some concerns in overall bias, 2 as having a high risk of bias [20,21], and 1 as having a low risk of bias [19] (Figure 2 [19-22,50-65]). Due to the nature and setting of the intervention, it was not possible to blind the patients or therapists delivering the intervention, leading to some concerns in the second domain (deviations from the intended intervention). There was a high risk of bias regarding the measurement of outcome because of insufficient information on blinded assessment [20,21]. Baseline between intervention groups had significant differences in 2 studies [64,65].

### Outcomes

#### Pain

**Figure 3:**
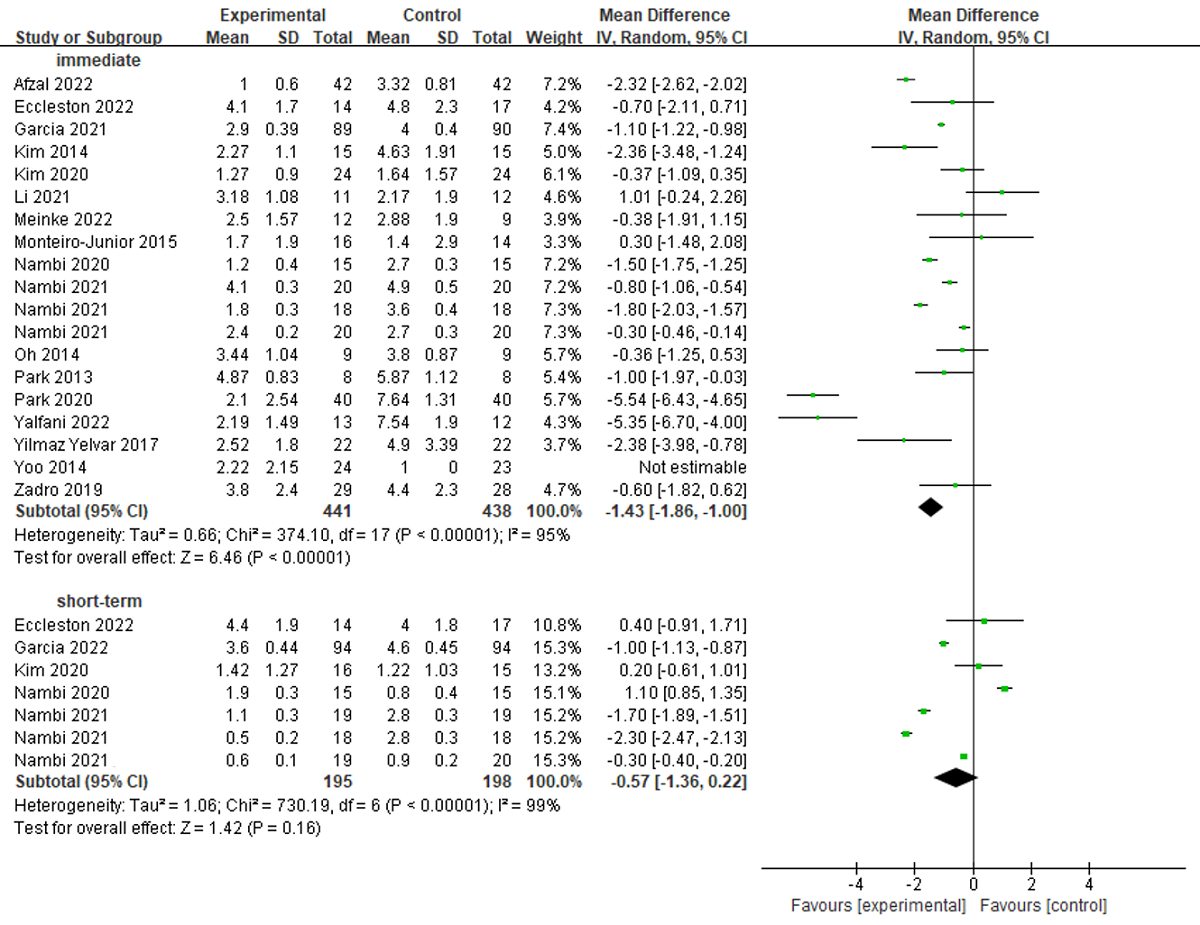
Forest plots for virtual reality–based training compared with controls in pain. IV: inverse variance.

All studies investigated the efficacy of virtual reality on pain using the Visual Analog Scale, Numerical Rating Scale, and Defense and Veterans Pain Rating Scale on a scale from 0 to 10. In total, 19 RCTs provided available data that were pooled into a meta-analysis (Figure 3 [19-22,50-60,62-65]). There were statistically significant differences between virtual reality–based training and conventional treatments for pain reduction in the immediate term (MD –1.43; 95% CI –1.86 to –1.00; *I*^2^=95%; *P*<.001) but not in the short term (MD –0.57; 95% CI –1.36 to 0.22; *I*^2^=99%; *P*=.16). Both did not reach an MCID at the level of 2.5 [66,67].

#### Pain-Related Fear

**Figure 4:**
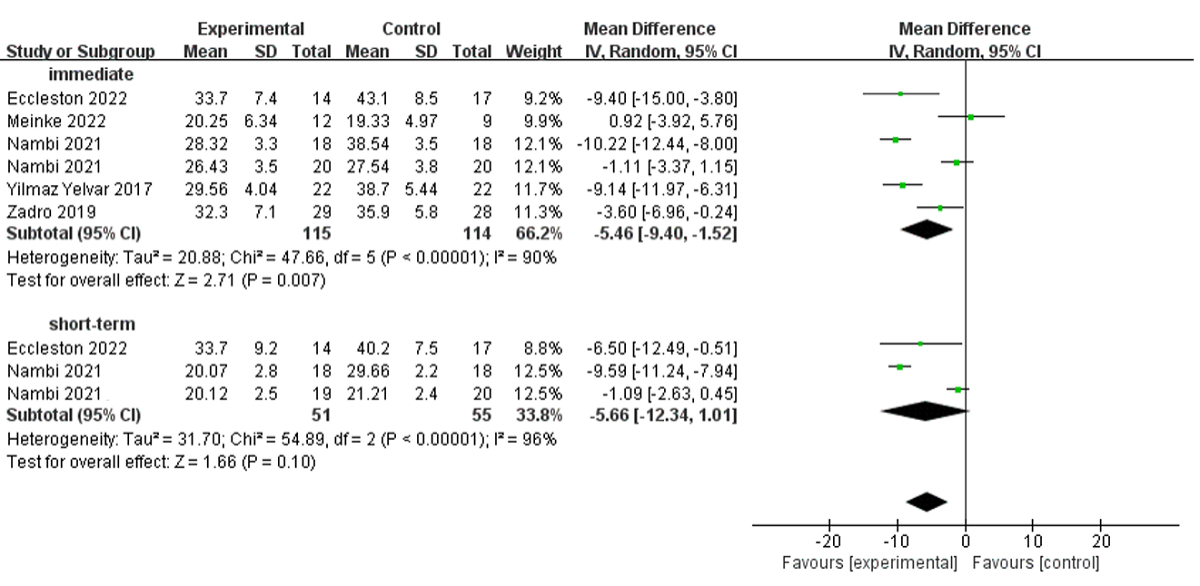
Forest plots for virtual reality–based training compared with controls in pain-related fear. IV: inverse variance.

In total, 6 studies investigated the efficacy of virtual reality–based training on pain-related fear using the Tampa Scale of Kinesiophobia (Figure 4 [50,52,53,63-65]). Virtual reality–based training showed significant improvements in individuals with chronic low back pain in the immediate term (MD –5.46; 95% CI –9.40 to –1.52; *I*^2^=90%; *P*=.007), while it did not show statistically significant differences in the short term (MD –5.66; 95% CI –12.34 to 1.01; *I*^2^=96%; *P*=.10). The difference in the immediate- and short-term performance was extremely close to an MCID at the level of 5.5 [68].

#### Disability

**Figure 5:**
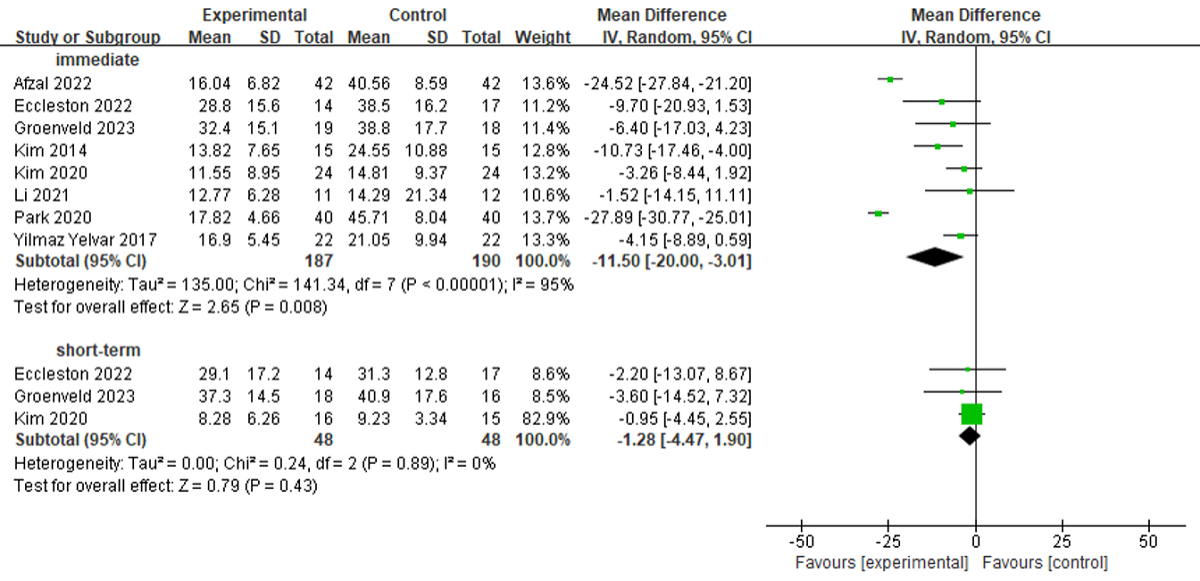
Forest plots for virtual reality–based training compared with controls in disability. IV: inverse variance.

In total, 8 studies investigated the efficacy of virtual reality–based training on disability using the ODI (Figure 5 [19,20,50,51,58,61,62,64]). There were statistically significant differences between virtual reality–based training and conventional treatments in terms of the ODI in the immediate term (MD –11.50; 95% CI –20.00 to –3.01; *I*^2^=95%; *P*=.008) but not in the short term (MD –1.28; 95% CI –4.47 to 1.90; *I*^2^=0%; *P*=.43). Only the immediate-term outcome did reach an MCID at the level of 10 [66,69].

### Quality of Evidence

The quality of evidence ranged from low to very low due to the risk of bias, imprecision, and inconsistency of included trials (Table 2). None were downgraded because of indirectness and publication bias.

**Table 2:** Summary of the findings and level of certainty using GRADE (Grading of Recommendations, Assessment, Development and Evaluation).

Time point and outcomes			Risk of bias		Inconsistency		Indirectness		Imprecision		Publication bias		Participants, n		RCT^a^, n		Mean difference (95% CI)		Certainty
**Immediate term**																			
	Pain	Serious^b^		Serious^c^		Not serious		Not serious		Not serious		879		19		–1.43 (–1.86 to –1.00)		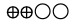 Low	
	Pain-related fear	Serious^b^		Serious^c^		Not serious		Serious^d^		Not serious		229		6		–5.46 (–9.40 to –1.52)		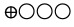 Very low	
	Disability	Serious^b^		Serious^c^		Not serious		Serious^d^		Not serious		377		8		–11.50 (–20.00 to –3.01)		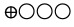 Very low	
**Short term**																			
	Pain-related fear	Serious^b^		Serious^c^		Not serious		Serious^d^		Not serious		106		3		–5.66 (–12.34 to 1.01)		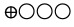 Very low	
	Pain	Serious^b^		Serious^c^		Not serious		Serious^d^		Not serious		393		7		–0.57 (–1.36 to 0.22)		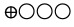 Very low	
	Disability	Serious^b^		Not serious		Not serious		Serious^d^		Not serious		96		3		–1.28 (–4.47 to 1.90)		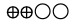 Low	

^a^RCT: randomized controlled trial.

^b^Downgrade due to the majority of trials rated as some concerns.

^c^Downgrade due to *I*^2^ statistics >50%.

^d^Downgrade due to pooled sample sizes <400.

## Discussion

### Principal Findings

This systematic review and meta-analysis, based on 20 RCTs, aimed to assess the available evidence on the efficacy of virtual reality–based training for people with chronic low back pain. The findings indicated that virtual reality–based training appeared to be an effective method of improving pain, pain-related fear, and disability immediately after intervention. However, current evidence failed to support the effectiveness of virtual reality–based training in the short term.

Virtual reality–based training has demonstrated efficacy in pain reduction for people with chronic low back pain [30,70]. However, our pooled results showed only a statistically significant reduction in pain immediately after interventions but not in the short term, which is consistent with previous studies [71-73]. In this study, there were no clinically significant differences between the immediate- and short-term performance. This may be explained by the fact that the virtual reality environment contributes to distraction from pain-related information [74,75]. However, once they gradually return to routines, the attention bias to pain might develop more often than being immersed in a virtual reality environment. This is supported by the finding that patients with chronic pain may less easily distract from the pain [76,77] due to higher levels of attention bias to pain [78].

Pain-related fear can activate the avoidance of movement, leading to the progression of disability [1]. In this study, we found that virtual reality–based training was superior to improve pain-related fear immediately after interventions, which is consistent with the effect on disability for individuals with chronic low back pain. It is worth noting that fear avoidance limits the opportunity to attune expectations to actual experiences, leading to long-term disability [79]. Consequently, with the alleviation of pain-related fear, individuals with chronic low back pain may be willing to pursue a positive experience, which is helpful to increase the level of physical activity. Although the large difference in the efficacy on pain-related fear in the short term indicated clinically important results but not statistically significant results, it should be interpreted cautiously due to the very low quality of evidence. Furthermore, the efficacy of virtual reality–based training on disability in the short term is also with low evidence. More high-quality RCTs are urgently needed for future research.

### Strengths and Limitations

This systematic review and meta-analysis conducted a thorough screening and search strategy in 6 important databases. Additionally, this study focused on RCTs to reinforce the evidence of pooled results. We also used the Cochrane Risk of Bias tool and the GRADE approach to rate the quality of the included studies and evidence, respectively. However, the potential limitations in this study need to be mentioned. First, high heterogeneity was presented in this meta-analysis due to the variety of sample size, intervention items, frequency, duration, and control items. Second, the study population varied from each other, especially age and gender. Third, there were only data on short-term outcomes (3 to 6 months after interventions), and no available data on the mid- or long-term (more than 6 months) effects of virtual reality–based training for participants with chronic low back pain. These findings indicated that more studies are required to strengthen the evidence.

### Implications for Clinical Practice and Future Research

Despite the low level of certainty, these findings may provide important implications for health care professionals. In clinical practice, the virtual reality–based training may be recommended for patients with chronic low back pain in order to distract and reduce the focus on pain. However, the persistence of efficacy needs to be considered when applying virtual reality–based training. Based on the present evidence in this study, it may be even better for immediate effects. Furthermore, the virtual reality–based training should be tailored to the needs and characteristics of patients in order to optimize the performance and maintain the efficacy. Future research should focus on different types of virtual reality, intervention parameters (eg, types, frequency, intensity, and time), and different population. These issues require further refinement through larger sample sizes, longer intervention duration, and longer follow-ups. In addition, it is urgent to explore the feasibility of virtual reality–based training in different socioeconomic contexts to ensure broader and cost-effective access to this type of intervention.

### Conclusions

In general, these findings support that virtual reality–based training is a promising treatment strategy for individuals with chronic low back pain immediately after interventions, especially to alleviate pain, pain-related fear, and disability. However, there is still not sufficient evidence to suggest that virtual reality–based training is effective in chronic low back pain in the short term. More high-quality RCTs are required to find short- and long-term benefits and to obtain more robust evidence.

## Appendix

Multimedia Appendix 1 PRISMA (Preferred Reporting Items for Systematic Reviews and Meta-Analyses) checklist.

Multimedia Appendix 2 Search strategy for PubMed, Embase, CINAHL, CENTRAL, and Web of Science.

## Abbreviations

GRADE: Grading of Recommendations, Assessment, Development and Evaluation
MCID: minimal clinically important difference
MD: mean difference
ODI: Oswestry Disability Index
PRISMA: Preferred Reporting Items for Systematic Reviews and Meta-Analyses
RCT: randomized controlled trial

## Data Availability

The data sets generated and analyzed during this study are available from the corresponding author on reasonable request.

## References

[1] Gatchel RJ, Peng YB, Peters ML, Fuchs PN, Turk DC (2007). The biopsychosocial approach to chronic pain: scientific advances and future directions. Psychol Bull.

[2] Mescouto K, Olson RE, Hodges PW, Setchell J (2022). A critical review of the biopsychosocial model of low back pain care: time for a new approach?. Disabil Rehabil.

[3] Kamper SJ, Apeldoorn AT, Chiarotto A, Smeets RJEM, Ostelo RWJG, Guzman J, van Tulder MW (2015). Multidisciplinary biopsychosocial rehabilitation for chronic low back pain: Cochrane systematic review and meta-analysis. BMJ.

[4] GBD 2019 Diseases and Injuries Collaborators (2020). Global burden of 369 diseases and injuries in 204 countries and territories, 1990-2019: a systematic analysis for the Global Burden of Disease Study 2019. Lancet.

[5] Chen S, Chen M, Wu X, Lin S, Tao C, Cao H, Shao Z, Xiao G (2022). Global, regional and national burden of low back pain 1990-2019: a systematic analysis of the Global Burden of Disease study 2019. J Orthop Translat.

[6] Hartvigsen J, Hancock MJ, Kongsted A, Louw Q, Ferreira ML, Genevay S, Hoy D, Karppinen J, Pransky G, Sieper J, Smeets RJ, Underwood M (2018). What low back pain is and why we need to pay attention. Lancet.

[7] Wertli MM, Eugster R, Held U, Steurer J, Kofmehl R, Weiser S (2014). Catastrophizing—a prognostic factor for outcome in patients with low back pain: a systematic review. Spine J.

[8] Synnott A, O'Keeffe M, Bunzli S, Dankaerts W, O'Sullivan P, O'Sullivan K (2015). Physiotherapists may stigmatise or feel unprepared to treat people with low back pain and psychosocial factors that influence recovery: a systematic review. J Physiother.

[9] O'Keeffe M, George SZ, O'Sullivan PB, O'Sullivan K (2019). Psychosocial factors in low back pain: letting go of our misconceptions can help management. Br J Sports Med.

[10] Buchbinder R, van Tulder M, Öberg Birgitta, Costa LM, Woolf A, Schoene M, Croft P (2018). Low back pain: a call for action. Lancet.

[11] Foster NE, Anema JR, Cherkin D, Chou R, Cohen SP, Gross DP, Ferreira PH, Fritz JM, Koes BW, Peul W, Turner JA, Maher CG (2018). Prevention and treatment of low back pain: evidence, challenges, and promising directions. Lancet.

[12] Bernstein IA, Malik Q, Carville S, Ward S (2017). Low back pain and sciatica: summary of NICE guidance. BMJ.

[13] Chou R, Deyo R, Friedly J, Skelly A, Hashimoto R, Weimer M, Fu R, Dana T, Kraegel P, Griffin J, Grusing S, Brodt ED (2017). Nonpharmacologic therapies for low back pain: a systematic review for an American College of Physicians Clinical Practice Guideline. Ann Intern Med.

[14] Miyamoto GC, Franco KFM, van Dongen JM, Franco YRDS, de Oliveira NTB, Amaral DDV, Branco ANC, da Silva ML, van Tulder MW, Cabral CMN (2018). Different doses of Pilates-based exercise therapy for chronic low back pain: a randomised controlled trial with economic evaluation. Br J Sports Med.

[15] Qaseem A, Wilt TJ, McLean RM, Forciea MA (2017). Noninvasive treatments for acute, subacute, and chronic low back pain: a clinical practice guideline from the American College of Physicians. Ann Intern Med.

[16] Saper RB, Lemaster C, Delitto A, Sherman KJ, Herman PM, Sadikova E, Stevans J, Keosaian JE, Cerrada CJ, Femia AL, Roseen EJ, Gardiner P, Gergen Barnett K, Faulkner C, Weinberg J (2017). Yoga, physical therapy, or education for chronic low back pain. Ann Intern Med.

[17] Hayden JA, Ellis J, Ogilvie R, Malmivaara A, van Tulder MW (2021). Exercise therapy for chronic low back pain. Cochrane Database Syst Rev.

[18] Powell KE, Paluch AE, Blair SN (2011). Physical activity for health: what kind? how much? how intense? on top of what?. Annu Rev Public Health.

[19] Afzal MW, Ahmad A, Mohseni Bandpei MA, Gilani SA, Hanif A, Waqas MS (2022). Effects of virtual reality exercises and routine physical therapy on pain intensity and functional disability in patients with chronic low back pain. J Pak Med Assoc.

[20] Kim SS, Min WK, Kim JH, Lee BH (2014). The effects of VR-based Wii Fit Yoga on physical function in middle-aged female LBP patients. J Phys Ther Sci.

[21] Park JH, Lee SH, Ko DS (2013). The effects of the Nintendo Wii exercise program on chronic work-related low back pain in industrial workers. J Phys Ther Sci.

[22] Yalfani A, Abedi M, Raeisi Z (2022). Effects of an 8-week virtual reality training program on pain, fall risk, and quality of life in elderly women with chronic low back pain: double-blind randomized clinical trial. Games Health J.

[23] Mirelman A, Maidan I, Deutsch JE (2013). Virtual reality and motor imagery: promising tools for assessment and therapy in Parkinson's disease. Mov Disord.

[24] Wiederhold BK, Soomro A, Riva G, Wiederhold MD (2014). Future directions: advances and implications of virtual environments designed for pain management. Cyberpsychol Behav Soc Netw.

[25] Matheve T, Bogaerts K, Timmermans A (2020). Virtual reality distraction induces hypoalgesia in patients with chronic low back pain: a randomized controlled trial. J Neuroeng Rehabil.

[26] Hoffman HG, Richards TL, Van Oostrom T, Coda BA, Jensen MP, Blough DK, Sharar SR (2007). The analgesic effects of opioids and immersive virtual reality distraction: evidence from subjective and functional brain imaging assessments. Anesth Analg.

[27] Kowatsch T, Lohse KM, Erb V, Schittenhelm L, Galliker H, Lehner R, Huang EM (2021). Hybrid ubiquitous coaching with a novel combination of mobile and holographic conversational agents targeting adherence to home exercises: four design and evaluation studies. J Med Internet Res.

[28] Thomas JS, France CR, Applegate ME, Leitkam ST, Walkowski S (2016). Feasibility and safety of a virtual reality dodgeball intervention for chronic low back pain: a randomized clinical trial. J Pain.

[29] Georgescu R, Fodor LA, Dobrean A, Cristea IA (2020). Psychological interventions using virtual reality for pain associated with medical procedures: a systematic review and meta-analysis. Psychol Med.

[30] Bordeleau M, Stamenkovic A, Tardif PA, Thomas J (2022). The use of virtual reality in back pain rehabilitation: a systematic review and meta-analysis. J Pain.

[31] Brea-Gómez B, Torres-Sánchez I, Ortiz-Rubio A, Calvache-Mateo A, Cabrera-Martos I, López-López L, Valenza MC (2021). Virtual reality in the treatment of adults with chronic low back pain: a systematic review and meta-analysis of randomized clinical trials. Int J Environ Res Public Health.

[32] Grassini S (2022). Virtual reality assisted non-pharmacological treatments in chronic pain management: a systematic review and quantitative meta-analysis. Int J Environ Res Public Health.

[33] Gumaa M, Rehan Youssef A (2019). Is virtual reality effective in orthopedic rehabilitation? a systematic review and meta-analysis. Phys Ther.

[34] Moher D, Liberati A, Tetzlaff J, Altman DG, PRISMA Group (2009). Preferred reporting items for systematic reviews and meta-analyses: the PRISMA statement. BMJ.

[35] Page MJ, McKenzie JE, Bossuyt PM, Boutron I, Hoffmann TC, Mulrow CD, Shamseer L, Tetzlaff JM, Akl EA, Brennan SE, Chou R, Glanville J, Grimshaw JM, Hróbjartsson A, Lalu MM, Li T, Loder EW, Mayo-Wilson E, McDonald S, McGuinness LA, Stewart LA, Thomas J, Tricco AC, Welch VA, Whiting P, Moher D (2021). The PRISMA 2020 statement: an updated guideline for reporting systematic reviews. BMJ.

[36] Boonstra AM, Schiphorst Preuper HR, Reneman MF, Posthumus JB, Stewart RE (2008). Reliability and validity of the visual analogue scale for disability in patients with chronic musculoskeletal pain. Int J Rehabil Res.

[37] Chapman JR, Norvell DC, Hermsmeyer JT, Bransford RJ, DeVine J, McGirt MJ, Lee MJ (2011). Evaluating common outcomes for measuring treatment success for chronic low back pain. Spine.

[38] Nassif TH, Hull A, Holliday SB, Sullivan P, Sandbrink F (2015). Concurrent validity of the defense and veterans pain rating scale in VA outpatients. Pain Med.

[39] Williamson A, Hoggart B (2005). Pain: a review of three commonly used pain rating scales. J Clin Nurs.

[40] Lundberg MKE, Styf J, Carlsson SG (2009). A psychometric evaluation of the Tampa Scale for Kinesiophobia—from a physiotherapeutic perspective. Physiother Theory Pract.

[41] Woby SR, Roach NK, Urmston M, Watson PJ (2005). Psychometric properties of the TSK-11: a shortened version of the Tampa Scale for Kinesiophobia. Pain.

[42] Fairbank JC, Pynsent PB (2000). The Oswestry Disability Index. Spine.

[43] Sterne JAC, Savović J, Page MJ, Elbers RG, Blencowe NS, Boutron I, Cates CJ, Cheng H, Corbett MS, Eldridge SM, Emberson JR, Hernán MA, Hopewell S, Hróbjartsson A, Junqueira DR, Jüni P, Kirkham JJ, Lasserson T, Li T, McAleenan A, Reeves BC, Shepperd S, Shrier I, Stewart LA, Tilling K, White IR, Whiting PF, Higgins JPT (2019). RoB 2: a revised tool for assessing risk of bias in randomised trials. BMJ.

[44] Guyatt GH, Oxman AD, Vist GE, Kunz R, Falck-Ytter Y, Alonso-Coello P, Schünemann HJ (2008). GRADE: an emerging consensus on rating quality of evidence and strength of recommendations. BMJ.

[45] Guyatt GH, Oxman AD, Kunz R, Brozek J, Alonso-Coello P, Rind D, Devereaux PJ, Montori VM, Freyschuss B, Vist G, Jaeschke R, Williams JW, Murad MH, Sinclair D, Falck-Ytter Y, Meerpohl J, Whittington C, Thorlund K, Andrews J, Schünemann HJ (2011). GRADE guidelines 6. rating the quality of evidence—imprecision. J Clin Epidemiol.

[46] Guyatt GH, Oxman AD, Vist G, Kunz R, Brozek J, Alonso-Coello P, Montori V, Akl EA, Djulbegovic B, Falck-Ytter Y, Norris SL, Williams JW, Atkins D, Meerpohl J, Schünemann HJ (2011). GRADE guidelines: 4. rating the quality of evidence—study limitations (risk of bias). J Clin Epidemiol.

[47] Guyatt GH, Oxman AD, Montori V, Vist G, Kunz R, Brozek J, Alonso-Coello P, Djulbegovic B, Atkins D, Falck-Ytter Y, Williams JW, Meerpohl J, Norris SL, Akl EA, Schünemann HJ (2011). GRADE guidelines: 5. rating the quality of evidence—publication bias. J Clin Epidemiol.

[48] Guyatt GH, Oxman AD, Kunz R, Woodcock J, Brozek J, Helfand M, Alonso-Coello P, Glasziou P, Jaeschke R, Akl EA, Norris S, Vist G, Dahm P, Shukla VK, Higgins J, Falck-Ytter Y, Schünemann HJ (2011). GRADE guidelines: 7. rating the quality of evidence—inconsistency. J Clin Epidemiol.

[49] Guyatt GH, Oxman AD, Kunz R, Woodcock J, Brozek J, Helfand M, Alonso-Coello P, Falck-Ytter Y, Jaeschke R, Vist G, Akl EA, Post PN, Norris S, Meerpohl J, Shukla VK, Nasser M, Schünemann HJ (2011). GRADE guidelines: 8. rating the quality of evidence—indirectness. J Clin Epidemiol.

[50] Eccleston C, Fisher E, Liikkanen S, Sarapohja T, Stenfors C, Jääskeläinen Satu K, Rice ASC, Mattila L, Blom T, Bratty JR (2022). A prospective, double-blind, pilot, randomized, controlled trial of an "embodied" virtual reality intervention for adults with low back pain. Pain.

[51] Li Z, Yu Q, Luo H, Liang W, Li X, Ge L, Zhang S, Li L, Wang C (2021). The effect of virtual reality training on anticipatory postural adjustments in patients with chronic nonspecific low back pain: a preliminary study. Neural Plast.

[52] Nambi G, Abdelbasset WK, Alrawaili SM, Alsubaie SF, Abodonya AM, Saleh AK (2021). Virtual reality or isokinetic training; its effect on pain, kinesiophobia and serum stress hormones in chronic low back pain: a randomized controlled trial. Technol Health Care.

[53] Nambi G, Abdelbasset WK, Alsubaie SF, Saleh AK, Verma A, Abdelaziz MA, Alkathiry AA (2021). Short-term psychological and hormonal effects of virtual reality training on chronic low back pain in soccer players. J Sport Rehabil.

[54] Nambi G, Abdelbasset WK, Elsayed SH, Alrawaili SM, Abodonya AM, Saleh AK, Elnegamy TE (2020). Comparative effects of isokinetic training and virtual reality training on sports performances in university football players with chronic low back pain-randomized controlled study. Evid Based Complement Alternat Med.

[55] Nambi G, Abdelbasset WK, Elsayed SH, Verma A, George JS, Saleh AK (2021). Eficiência clínica e física de jogos de realidade virtual em jogadores de futebol com dor lombar. Clinical and physical efficiency of virtual reality games in soccer players with low back pain. Rev Bras Med Esporte.

[56] Oh HW, Lee MG, Jang JY, Jin JJ, Cha JY, Jin YY, Jee YS (2014). Time-effects of horse simulator exercise on psychophysiological responses in men with chronic low back pain. IES.

[57] Monteiro-Junior RS, de Souza CP, Lattari E, Rocha NBF, Mura G, Machado S, da Silva EB (2015). Wii-workouts on chronic pain, physical capabilities and mood of older women: a randomized controlled double blind trial. CNS Neurol Disord Drug Targets.

[58] Park S, Park S, Min S, Kim CJ, Jee YS (2020). A randomized controlled trial investigating the effects of equine simulator riding on low back pain, morphological changes, and trunk musculature in elderly women. Medicina (Kaunas).

[59] Yoo JH, Kim SE, Lee MG, Jin JJ, Hong J, Choi YT, Kim MH, Jee YS (2014). The effect of horse simulator riding on visual analogue scale, body composition and trunk strength in the patients with chronic low back pain. Int J Clin Pract.

[60] Garcia LM, Birckhead BJ, Krishnamurthy P, Sackman J, Mackey IG, Louis RG, Salmasi V, Maddox T, Darnall BD (2021). An 8-week self-administered at-home behavioral skills-based virtual reality program for chronic low back pain: double-blind, randomized, placebo-controlled trial conducted during COVID-19. J Med Internet Res.

[61] Groenveld TD, Smits MLM, Knoop J, Kallewaard JW, Staal JB, de Vries M, van Goor H (2023). Effect of a behavioral therapy-based virtual reality application on quality of life in chronic low back pain. Clin J Pain.

[62] Kim T, Lee J, Oh S, Kim S, Yoon B (2020). Effectiveness of simulated horseback riding for patients with chronic low back pain: a randomized controlled trial. J Sport Rehabil.

[63] Meinke A, Peters R, Knols RH, Swanenburg J, Karlen W (2022). Feedback on trunk movements from an electronic game to improve postural balance in people with nonspecific low back pain: pilot randomized controlled trial. JMIR Serious Games.

[64] Yilmaz Yelvar GD, Çırak Y, Dalkılınç M, Parlak Demir Y, Guner Z, Boydak A (2017). Is physiotherapy integrated virtual walking effective on pain, function, and kinesiophobia in patients with non-specific low-back pain? randomised controlled trial. Eur Spine J.

[65] Zadro JR, Shirley D, Simic M, Mousavi SJ, Ceprnja D, Maka K, Sung J, Ferreira P (2019). Video-game-based exercises for older people with chronic low back pain: a randomized controlledtable trial (GAMEBACK). Phys Ther.

[66] Ostelo RWJG, de Vet HCW (2005). Clinically important outcomes in low back pain. Best Pract Res Clin Rheumatol.

[67] van der Roer N, Ostelo RWJG, Bekkering GE, van Tulder MW, de Vet HCW (2006). Minimal clinically important change for pain intensity, functional status, and general health status in patients with nonspecific low back pain. Spine.

[68] Monticone M, Ambrosini E, Rocca B, Foti C, Ferrante S (2016). Responsiveness of the Tampa Scale of Kinesiophobia in Italian subjects with chronic low back pain undergoing motor and cognitive rehabilitation. Eur Spine J.

[69] Ostelo RWJG, Deyo RA, Stratford P, Waddell G, Croft P, Von Korff M, Bouter LM, de Vet HC (2008). Interpreting change scores for pain and functional status in low back pain: towards international consensus regarding minimal important change. Spine.

[70] Nagpal AS, Raghunandan A, Tata F, Kibler D, McGeary D (2022). Virtual reality in the management of chronic low back pain: a scoping review. Front Pain Res (Lausanne).

[71] House G, Burdea G, Grampurohit N, Polistico K, Roll D, Damiani F, Hundal J, Demesmin D (2016). A feasibility study to determine the benefits of upper extremity virtual rehabilitation therapy for coping with chronic pain post-cancer surgery. Br J Pain.

[72] Sato K, Fukumori S, Matsusaki T, Maruo T, Ishikawa S, Nishie H, Takata K, Mizuhara H, Mizobuchi S, Nakatsuka H, Matsumi M, Gofuku A, Yokoyama M, Morita K (2010). Nonimmersive virtual reality mirror visual feedback therapy and its application for the treatment of complex regional pain syndrome: an open-label pilot study. Pain Med.

[73] Ortiz-Catalan M, Guðmundsdóttir Rannveig A, Kristoffersen MB, Zepeda-Echavarria A, Caine-Winterberger K, Kulbacka-Ortiz K, Widehammar C, Eriksson K, Stockselius A, Ragnö Christina, Pihlar Z, Burger H, Hermansson L (2016). Phantom motor execution facilitated by machine learning and augmented reality as treatment for phantom limb pain: a single group, clinical trial in patients with chronic intractable phantom limb pain. Lancet.

[74] Kohl A, Rief W, Glombiewski JA (2013). Acceptance, cognitive restructuring, and distraction as coping strategies for acute pain. J Pain.

[75] Verhoeven K, Crombez G, Eccleston C, Van Ryckeghem DML, Morley S, Van Damme S (2010). The role of motivation in distracting attention away from pain: an experimental study. Pain.

[76] Van Ryckeghem DM, Van Damme S, Eccleston C, Crombez G (2018). The efficacy of attentional distraction and sensory monitoring in chronic pain patients: a meta-analysis. Clin Psychol Rev.

[77] Vlaeyen JWS, Morley S, Crombez G (2016). The experimental analysis of the interruptive, interfering, and identity-distorting effects of chronic pain. Behav Res Ther.

[78] Todd J, van Ryckeghem DML, Sharpe L, Crombez G (2018). Attentional bias to pain-related information: a meta-analysis of dot-probe studies. Health Psychol Rev.

[79] Crombez G, Eccleston C, Van Damme S, Vlaeyen JWS, Karoly P (2012). Fear-avoidance model of chronic pain: the next generation. Clin J Pain.

