# Supplementary material for: Virtual Reality–Based Training in Chronic Low Back Pain: Systematic Review and Meta-Analysis of Randomized Controlled Trials
Source: J Med Internet Res. 2024 Feb 26;26:e45406. doi: 10.2196/45406 (PMC10928528; doi:10.2196/45406)
Supplement: Multimedia Appendix 2 [file jmir_v26i1e45406_app2.docx]

**Search strategy for Pubmed <January 2024>**

#1 (((((((Virtual Reality Exposure Therapy) OR (Virtual Reality Immersion Therapy[Title/Abstract])) OR (Virtual Reality Therapy[Title/Abstract])) OR (Reality Therapies, Virtual[Title/Abstract])) OR (Reality Therapy, Virtual[Title/Abstract])) OR (Therapies, Virtual Reality[Title/Abstract])) OR (Therapy, Virtual Reality[Title/Abstract])) OR (Virtual Reality Therapies[Title/Abstract]) 1679

#2 (((((((((((Virtual Reality) OR (Reality, Virtual[Title/Abstract]) OR (Virtual Reality, Educational[Title/Abstract])) OR (Educational Virtual Realities[Title/Abstract])) OR (Educational Virtual Reality[Title/Abstract])) OR (Reality, Educational Virtual[Title/Abstract])) OR (Virtual Realities, Educational[Title/Abstract])) OR (Virtual Reality, Instructional[Title/Abstract])) OR (Instructional Virtual Realities[Title/Abstract])) OR (Instructional Virtual Reality[Title/Abstract])) OR (Realities, Instructional Virtual[Title/Abstract])) OR (Reality, Instructional Virtual[Title/Abstract])) OR (Virtual Realities, Instructional[Title/Abstract]) 22007

#3 (((((((((((Exergaming) OR (Active-Video Gaming[Title/Abstract])) OR (Active Video Gaming[Title/Abstract])) OR (Active-Video Gamings[Title/Abstract])) OR (Gaming, Active-Video[Title/Abstract])) OR (Gamings, Active-Video[Title/Abstract])) OR (Virtual Reality Exercise[Title/Abstract])) OR (Exercise, Virtual Reality[Title/Abstract])) OR (Exercises, Virtual Reality[Title/Abstract])) OR (Virtual Reality Exercises[Title/Abstract])) OR (Exergames[Title/Abstract])) OR (Exergame[Title/Abstract]) 1686

#4 (((((((((((((((((((((((((((((Low Back Pain) OR (Back Pain, Low[Title/Abstract])) OR (Back Pains, Low[Title/Abstract])) OR (Low Back Pains[Title/Abstract])) OR (Pain, Low Back[Title/Abstract])) OR (Pains, Low Back[Title/Abstract])) OR (Lumbago[Title/Abstract])) OR (Lower Back Pain[Title/Abstract])) OR (Back Pain, Lower[Title/Abstract])) OR (Back Pains, Lower[Title/Abstract])) OR (Lower Back Pains[Title/Abstract])) OR (Pain, Lower Back[Title/Abstract])) OR (Pains, Lower Back[Title/Abstract])) OR (Low Back Ache[Title/Abstract])) OR (Ache, Low Back[Title/Abstract])) OR (Aches, Low Back[Title/Abstract])) OR (Back Ache, Low[Title/Abstract])) OR (Back Aches, Low[Title/Abstract])) OR (Low Back Aches[Title/Abstract])) OR (Low Backache[Title/Abstract])) OR (Backache, Low[Title/Abstract])) OR (Backaches, Low[Title/Abstract])) OR (Low Backaches[Title/Abstract])) OR (Low Back Pain, Postural[Title/Abstract])) OR (Postural Low Back Pain[Title/Abstract])) OR (Low Back Pain, Posterior Compartment[Title/Abstract])) OR (Low Back Pain, Recurrent[Title/Abstract])) OR (Recurrent Low Back Pain[Title/Abstract])) OR (Low Back Pain, Mechanical[Title/Abstract])) OR (Mechanical Low Back Pain[Title/Abstract]) 52732

#5 #1 OR #2 OR #3 23259

#6 #4 AND #5 81

**Search strategy for Embase < January 2024>**

#1 low AND ('back'/exp OR back) AND ('pain'/exp OR pain) 91162

#2 virtual AND reality 36531

#3 virtual reality exposure therapy 1148

#4 exergaming 860

#5 virtual AND reality AND exercise 2227

#6 #2 OR #3 OR #4 OR #5 37145

#7 #1 AND #6 139

**Search strategy for CINAHL <January 2024>**

S1 (((((((Virtual Reality Exposure Therapy) OR (Virtual Reality Immersion Therapy)) OR (Virtual Reality Therapy)) OR (Reality Therapies, Virtual)) OR (Reality Therapy, Virtual)) OR (Therapies, Virtual Reality)) OR (Therapy, Virtual Reality)) OR (Virtual Reality Therapies) 810

S2 (((((((((((Virtual Reality) OR (Reality, Virtual) OR (Virtual Reality, Educational)) OR (Educational Virtual Realities)) OR (Educational Virtual Reality)) OR (Reality, Educational Virtual)) OR (Virtual Realities, Educational)) OR (Virtual Reality, Instructional)) OR (Instructional Virtual Realities)) OR (Instructional Virtual Reality)) OR (Realities, Instructional Virtual)) OR (Reality, Instructional Virtual)) OR (Virtual Realities, Instructional) 8050

S3 (((((((((((Exergaming) OR (Active-Video Gaming)) OR (Active Video Gaming)) OR (Active-Video Gamings)) OR (Gaming, Active-Video)) OR (Gamings, Active-Video)) OR (Virtual Reality Exercise)) OR (Exercise, Virtual Reality)) OR (Exercises, Virtual Reality)) OR (Virtual Reality Exercises)) OR (Exergames)) OR (Exergame) 599

S4 (((((((((((((((((((((((((((((Low Back Pain) OR (Back Pain, Low)) OR (Back Pains, Low)) OR (Low Back Pains)) OR (Pain, Low Back)) OR (Pains, Low Back)) OR (Lumbago)) OR (Lower Back Pain)) OR (Back Pain, Lower)) OR (Back Pains, Lower)) OR (Lower Back Pains)) OR (Pain, Lower Back)) OR (Pains, Lower Back)) OR (Low Back Ache)) OR (Ache, Low Back)) OR (Aches, Low Back)) OR (Back Ache, Low)) OR (Back Aches, Low)) OR (Low Back Aches)) OR (Low Backache)) OR (Backache, Low)) OR (Backaches, Low)) OR (Low Backaches)) OR (Low Back Pain, Postural)) OR (Postural Low Back Pain)) OR (Low Back Pain, Posterior Compartment)) OR (Low Back Pain, Recurrent)) OR (Recurrent Low Back Pain)) OR (Low Back Pain, Mechanical)) OR (Mechanical Low Back Pain) 20555 8338

S5 S1 OR S2 OR S3 8447

S6 S4 AND S5 41

**Search strategy for Cochrane Central Register of Controlled Trials <January 2024>**

1 low back pain.mp. or exp Low Back Pain/ 13468

2 virtual reality.mp. or exp Virtual Reality/ 6400

3 virtual reality exposure therapy.mp. or exp Virtual Reality Exposure Therapy/ 496

4 virtual reality exercise.mp. or exp Exergaming/ 136

5 2 or 3 or 4 6446

6 1 and 5 94

**Search strategy for Web of science <January 2024>**

#1 (((TS=(virtual reality)) OR TS=(virtual reality exposure therapy)) OR TS=(virtual reality exercise)) OR TS=(exergaming) 240455

#2 TS=(low back pain) 104687

#3 #1 AND #2 211
